# The McGill Thyroid Nodule Score’s (MTNS+) role in the investigation of thyroid nodules with benign ultrasound guided fine needle aspiration biopsies: a retrospective review

**DOI:** 10.1186/s40463-016-0141-7

**Published:** 2016-05-04

**Authors:** Sarah Khalife, Sarah Bouhabel, Veronique-Isabelle Forest, Michael P. Hier, Louise Rochon, Michael Tamilia, Richard J. Payne

**Affiliations:** Department of Otolaryngology-Head and Neck Surgery, McGill University Health Centre, 1001 Boulevard Decarie, Montreal, QC Canada; Department of Otolaryngology-Head and Neck Surgery, Sir Mortimer B. Davis-Jewish General Hospital, McGill University, 3755 Côte Ste-Catherine Road, Montreal, QC Canada; Department of Pathology, Sir Mortimer B. Davis-Jewish General Hospital, McGill University, 3755 Côte Ste-Catherine Road, Montreal, QC Canada; Department of Endocrinology and Metabolism, Sir Mortimer B. Davis-Jewish General Hospital, McGill University, 3755 Côte Ste-Catherine Road, Montreal, QC Canada

**Keywords:** McGill Thyroid Nodule Score, Thyroid cancer, Ultrasound-guided fine needle aspiration biopsy, Benign nodule, Bethesda II, Nodule size

## Abstract

**Background:**

Ultrasound guided fine needle aspiration (USFNA) biopsies of thyroid nodules sometimes create a decision-making dilemma for surgeons as they may yield falsely benign results. The McGill Thyroid Nodule Score + (MTNS+) was developed to aid in clinical guidance regarding the management of patients with these USFNA results. The aim of this study was to assess the MTNS+ as a clinical tool in patients with benign preoperative thyroid nodule USFNAs and to analyze the relationship between nodule size and malignancy in these patients.

**Methods:**

We conducted a retrospective chart review of 1312 patients who underwent thyroidectomies between 2010 and 2015 at the McGill University Teaching Hospitals. Patients with Bethesda II (benign) USFNA results, calculated MTNS+, and nodule size evaluated on ultrasound were included in the study. The false-negative rate was calculated, and MTNS+ and nodule size were each compared to final pathology results. Binary logistic regression was used for statistical analysis.

**Results:**

Of the 1312 patients, 101 met the inclusion criteria and together had an average MTNS+ score of 6.83, which corresponds to a predicted malignancy rate between 25 and 33 %. Final pathology revealed malignancy in 16 (15.8 %) subjects. The average MTNS+ of patients with malignant nodules on surgical pathology was 8.25, while that of patients with benign nodules was 6.56.

Patients with nodule size 1–1.9 cm (a) and 2–2.9 cm (b) each had an equal rate of malignancy of 2.97 % (*n* = 3), nodule size 3–3.9 cm (c) had a rate of 1.98 % (*n* = 2), and nodule size ≥4 cm (d) a rate of 7.92 % (*n* = 8).

**Conclusion:**

The rate of malignancy (15.8 %) is higher than expected when reviewing the risk of malignancy in nodules considered as Bethesda class 2. On the other hand, the rate is lower than the 25–33 % predicted by the MTNS+. We also found a higher malignancy rate for nodules above 4 cm in size, but size was a poor predictor of malignancy when used alone. Therefore, while the MTNS+ may be helpful at helping to identify USFNAs that are incorrectly classified as benign, the percentage risk of malignancy is lower than expected.

## Background

The incidence of thyroid cancer has been significantly and rapidly increasing in Canada over the past 30 years [1, 2]. Ultrasound-guided fine needle aspiration (USFNA) biopsy is currently considered the gold standard for the assessment and management of thyroid nodules. While it is undeniable that USFNAs provide very important information as to the nature of the nodule, they may yield a falsely benign result in 5 % of cases [3, 4]. Given the potential health implications of misclassifying malignant thyroid nodules as benign, it is necessary to find a complementary clinical tool to evaluate the risk of malignancy and properly select patients who require surgery.

Previous studies [1, 5, 6] advocate the McGill Thyroid Nodule Score + (MTNS+), as a valuable scoring-system used to accurately determine a patient’s overall risk of malignancy. The MTNS+ is based on 23 known thyroid cancer risk factors, which are tabulated and then assigned a percentage risk of malignancy (Table 1) [6].

**Table 1:**
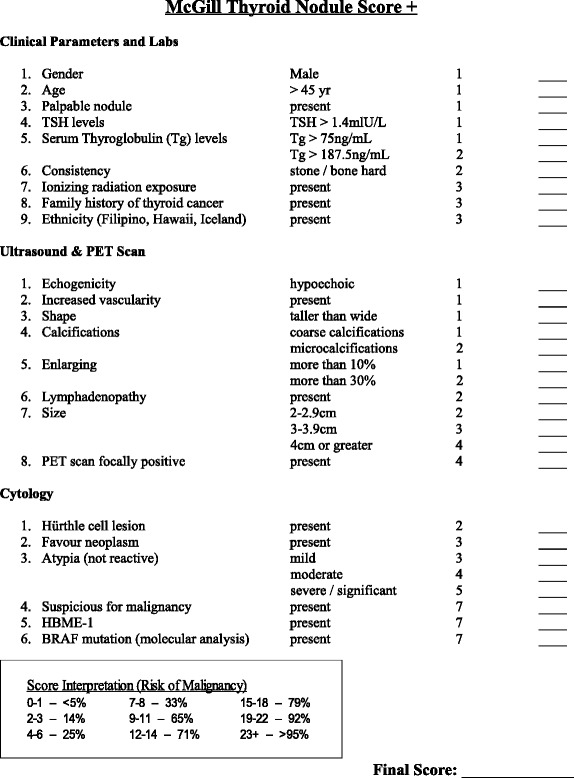
McGill Thyroid Nodule Score Plus (MTNS+) Scoring Template

*TSH* Thyroid stimulating hormone. *Tg* Thyroglobulin. *PET* Positron emission tomography

This study aims to assess the accuracy of the MTNS+ in predicting the risk of malignancy of thyroid nodules with benign USFNAs. The association between nodule size and malignancy rate is also evaluated. Findings could reduce the proportion of missed malignancies and improve management of patients with thyroid nodules.

## Methods

Multi-center ethics review approval was obtained from Research Ethics Committees at the Jewish General Hospital (JGH) and the McGill University Health Centre (MUHC).

We conducted a retrospective chart review of 1312 patients who underwent thyroidectomies at the McGill University Teaching Hospitals between January 2010 and March 2015. 101 patients (7.7 %) met inclusion criteria that consisted of having Bethesda II (benign) USFNA biopsy results, pre-operative calculated MTNS+ scores and recorded nodule size on ultrasound. We excluded patients with unavailable pre-operative MTNS+, USFNA and nodule size results. All USFNA samples were obtained by endocrinologists, thyroid surgeons, or radiologists. Collected information on the MTNS+ was based on a scoring sheet that has been regularly filled out over many years when a patient consults the doctor. Positron emission tomography (PET) scan and BRAF mutation are the two MTNS+ variables that are not routinely measured.

MTNS+ and thyroid nodule size on ultrasound were each compared to final post-operative pathology classified as either benign or malignant. Papillary microcarcinomas with extrathyroidal extension (ETE) were considered malignant due to their unpredictable aggressive behaviour when associated with ETE [6, 7]. All other papillary microcarcinomas were considered benign, as they usually progress indolently [6, 7]. Using USFNA allowed us to ensure malignancy was assigned to the ipsilateral nodule that was biopsied, thus avoiding falsely assigning malignancy to benign nodules in patients with multinodular thyroid glands. The malignancy rate, the mean MTNS+, and the standard deviations were calculated for the group of patients with Bethesda II cytology, as well as within each subgroup: malignant and benign on surgical pathology results. Statistical analysis was done using Binary Logistic Regression for MTNS+ when compared to malignancy rates. Nodule size was separated into four categories: a) 1–1.9 cm, b) 2–2.9 cm, c) 3–3.9 cm, and d) ≥4 cm. Malignancy rates were calculated for each category and a receiver operating characteristic (ROC) curve was also computed for malignancy and nodule size as a continuous variable. Bivariate regression analysis and Spearman’s correlations were run to compare MTNS+ and nodule size.

## Results

Of the 1312 charts reviewed, 101 patients (7.7 %) met inclusion criteria. Patient age ranged from 23 to 85, with a mean of 52 years old. There was a 6-fold female predominance with a male to female ratio of 14:87. Final pathology revealed malignancy in 16 subjects (15.8 %), while 85 (84.2 %) were benign. One of the 16 patients included in the malignant group had a microcarcinoma with ETE on final pathology. In this selected patient population, MTNS+ scores ranged from 1 to 18, with a mean value and standard deviation (SD) of 6.83 ± 2.31.

### MTNS+ and malignancy

**Fig. 1:**
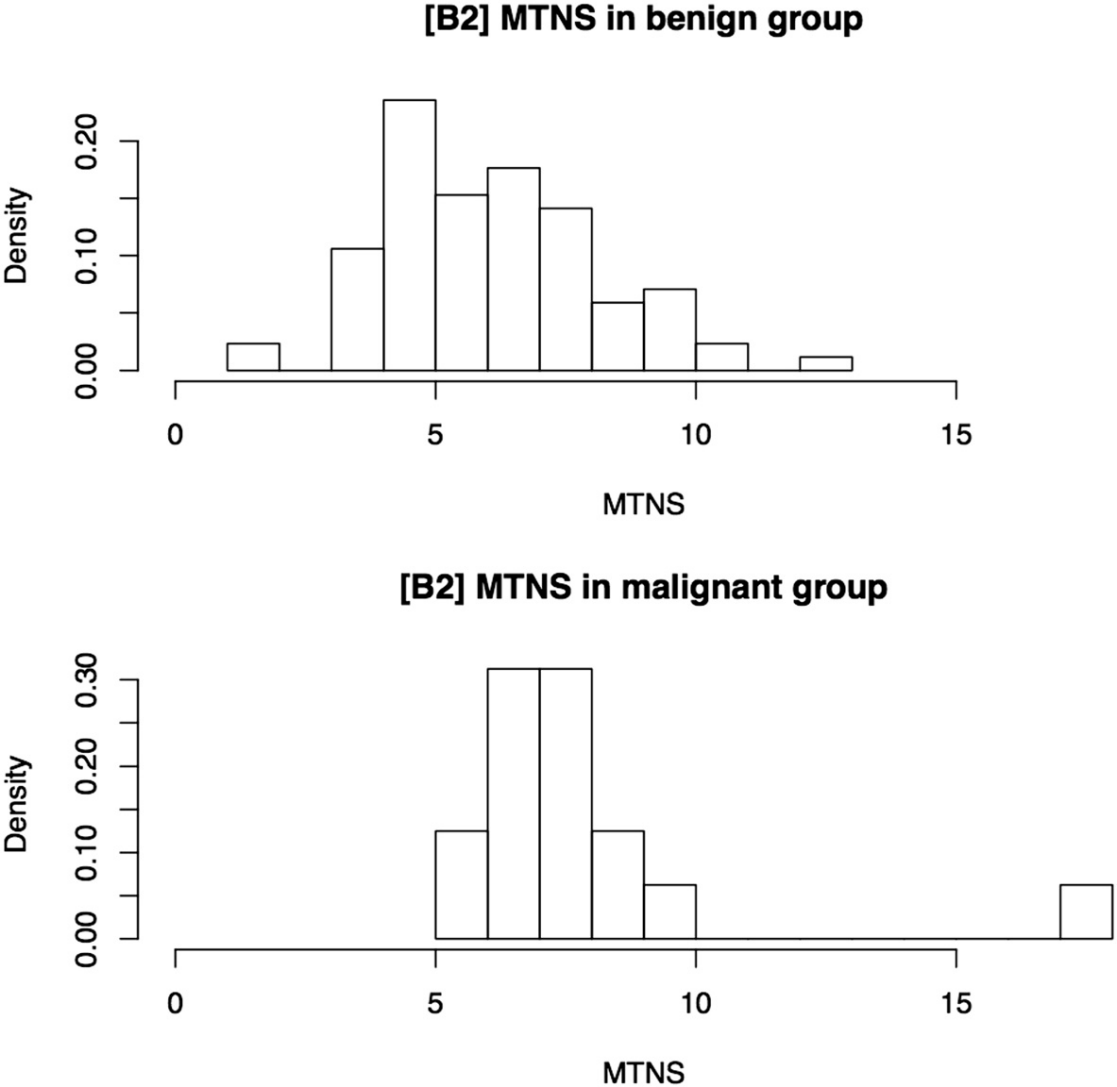
Frequency distribution of pre-operative MTNS+ in 101 thyroidectomy patients with Bethesda II USFNAs. Distribution of MTNS+ scores are displayed for thyroid nodules with: Top) Benign pathology on surgical biopsy, Bottom) Malignant pathology on surgical biopsy. MTNS+: McGill Thyroid Nodule Score Plus. USFNA: Ultrasound guided fine needle aspiration

Benign and malignant rates and densities for each MTNS+ are presented in Table 2 and Fig. 1, respectively. Amongst the 101 patients included in the study, all malignant nodules had an MTNS+ equal to or above 5, while benign nodules had MTNS+ values as low as 1. Average MTNS+ of patients with malignant pathology (8.25 ± 2.86 [95 % CI 6.72, 9.78]) was higher than MTNS+ of patients in the benign group (6.56 ± 2.11 [95 % CI 6.11, 7.02]) (Table 3). The MTNS+ mean difference between malignant and benign groups was 1.69 with 95 % confidence interval (CI) [0.11, 3.26].

**Table 2:** Distribution of benign USFNA MTNS+ scores and their associated surgical pathology diagnosis

MTNS	Total (n)	Benign n (%)	Malignant n (%)
1	1	1 (1.0)	0 (0.0)
2	1	1 (1.0)	0 (0.0)
3	0	0 (0.0)	0 (0.0)
4	9	9 (8.9)	0 (0.0)
5	21	20 (19.8)	1 (1.0)
6	14	13 (12.9)	1 (1.0)
7	20	15 (14.9)	5 (5.0)
8	17	12 (11.9)	5 (5.0)
9	7	5 (5.0)	2 (2.0)
10	7	6 (5.9)	1 (1.0)
11	2	2 (2.0)	0 (0.0)
12	0	0 (0.0)	0 (0.0)
13	1	1 (1.0)	0 (0.0)
14	0	0 (0.0)	0 (0.0)
15	0	0 (0.0)	0 (0.0)
16	0	0 (0.0)	0 (0.0)
17	0	0 (0.0)	0 (0.0)
18	1	0 (0.0)	1 (1.0)
Total	101	85 (84.2)	16 (15.8)

*MTNS+* McGill Thyroid Nodule Score Plus. *USFNA* Ultrasound guided fine needle aspiration

**Table 3:** Distribution of MTNS+ scores within benign and malignant thyroid nodule categories confirmed on surgical pathology

	Bethesda II Thyroid Nodules (*N* = 101)									
	Benign (84.2 %)					Malignant (15.8 %)				
	N	Mean	StdDev	Min	Max	N	Mean	StdDev	Min	Max
MTNS	85	6.56	2.11	1	13	16	8.25	2.86	5	18

*MTNS+* McGill Thyroid Nodule Score Plus. *StdDev* standard deviation

A positive association between MTNS+ and malignancy was confirmed by logistic regression analysis with an odds ratio of 1.34 [95 % CI 1.05, 1.71].

### MTNS+ and nodule size

Largest nodule size on ultrasound ranged between 1 and 8.9 cm with a mean and standard deviation of 4.13 cm ±1.53. A positive correlation between MTNS+ and nodule size was shown by Spearman’s correlation with an “r” coefficient value of 0.146 [95 % CI -0.05, 0.33]. The MTNS+ odds ratio was 1.52 [95 % CI 1.130, 2.044] when adjusted for nodule size through bivariate regression analysis. Patients with nodule size 1–1.9 cm had an OR of 16.2 [95 % CI 1.83, 143.427], those with nodule size 2–2.9 cm had an OR of 4.387 [95 % CI 0.806, 23.876], and nodule size 3–3.9 cm an OR of 0.341 [95 % CI 0.063, 1.832] when compared to nodules above 4 cm in size.

### Nodule size and malignancy

**Fig. 2:**
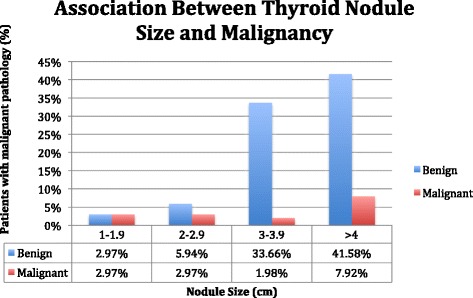
Association between thyroid nodule size and surgical pathology diagnosis in patients with benign USFNA biopsies. Nodule sizes on pre-operative ultrasounds were subdivided into categories a) 1–1.9 cm, b) 2–2.9 cm, c) 3–3.9 cm, d) ≥4 cm. USFNA: Ultrasound guided fine needle aspiration

**Fig. 3:**
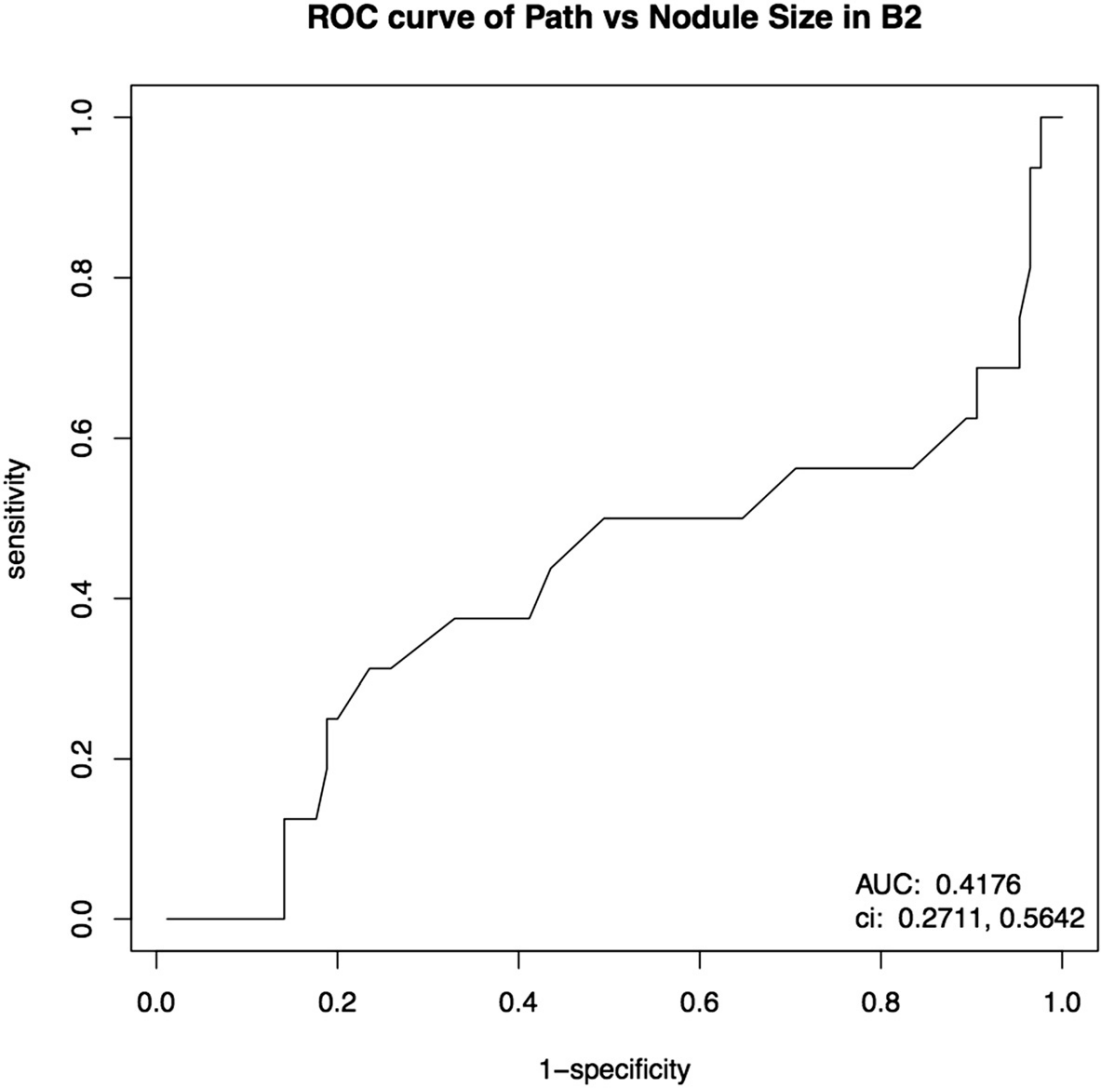
Receiver operating characteristic curve for thyroid nodule size and risk of malignancy. Nodule size was determined by pre-operative ultrasound and malignancy was confirmed by post-operative pathology

Benign and malignancy rates according to nodule size categories (cm) are presented in Table 4. Average nodule size was similar in patients with malignant and benign pathology (3.67 cm ±1.60, and 4.23 cm ±1.51, respectively). Patients with nodule size 1–1.9 cm (a) had a rate of malignancy of 2.97 % (*n* = 3), nodule size 2-2.9 cm (b) a rate of 2.97 % (*n* = 3), nodule size 3–3.9 cm (c) a rate of 1.98 % (*n* = 2), and nodule size ≥4 cm (d) a malignancy rate of 7.92 % (*n* = 8) (Fig. 2). ROC curve for nodule size and malignancy rate is presented in Fig. 3.

**Table 4:** Association of benign USFNA nodule size and their corresponding surgical pathology diagnosis

Nodule Size (cm)	Total n (%)	Benign n (%)	Malignant n (%)
a) 1–1.9	6 (5.94)	3 (2.97)	3 (2.97)
b) 2–2.9	9 (8.91)	6 (5.94)	3 (2.97)
c) 3–3.9	36 (35.64)	34 (33.66)	2 (1.98)
d) ≥4	50 (49.50)	42 (41.58)	8 (7.92)
Total	101 (100)	85 (84.16)	16 (15.84)

Nodule sizes were determined pre-operatively by ultrasound and were subdivided into categories a) 1–1.9 cm, b) 2–2.9 cm, c) 3–3.9 cm, d) ≥4 cm

*USFNA* Ultrasound guided fine needle aspiration

## Discussion

Approximately 60 % of all performed USFNAs are Bethesda II (benign) on cytology [8] and should ideally result in a relief for patients, surgeons, and clinicians, in terms of treatment and prognosis. However, the 5 % false-negative rate of benign USFNAs [3, 9] cannot be disregarded. This is why the American Thyroid Association (ATA), the American Association of Clinical Endocrinologists (AACE) and the European Thyroid Association (ETA) suggest close observation of patients with follow-up ultrasounds 6–18 months later, and repeat USFNA if nodule growth is seen [3, 10].

Despite these recommendations, researchers continue to challenge the falsely benign rate stated by the Bethesda Classification [2, 9–11]. The 15.8 % rate of malignancy identified in our specific series is substantially higher than that predicted by Bethesda and ATA guidelines for benign USFNAs. Yet, it is consistent with the higher rates reported throughout literature, reaching values as high as 24.2 % [2, 5, 10–12]. This wide range may be explained in part by variations in technique, expertise and subjective interpreting differences between pathologists reading the slides and physicians performing ultrasounds and USFNAs in different institutions. Nevertheless, the difference remains significant. With this scientific scepticism, benign USFNA results will continue to pose a dilemma for the thyroid surgeon, particularly when known risk factors such as suspicious ultrasound findings or exposure to ionizing radiation are present.

Several studies recommend considering both cytology and suspicious ultrasound findings before making treatment decisions [10, 12]. Interestingly, Chernyavsky et al. found that a remarkable 90 % of falsely benign USFNAs (10.2 %) in their series had suspicious characteristics on ultrasound [10]. They go on to suggest repeat USFNA in these cases, in support of several other publications [10, 12, 13].

This is the reason that an evidence-based scoring system such as the MTNS+ assists in the pre-operative assessment of thyroid nodules. Its use could allow for earlier therapeutic decision-making. The MTNS+ has been shown to be an accurate tool for predicting the risk of malignancy [1, 6]. This 23-parameter summative tool (Table 1) proposed by Sands et al. and then modified in 2013 by Scheffler et al., was developed in order to help physicians formulate the management of thyroid nodules [1, 6]. It takes into consideration multiple evidence-based risk factors such as clinical factors (family history, exposure to radiation), worrisome ultrasound features, as well as cytology results. Combined into a single score, they enable a comprehensive identification of thyroid nodules that are more likely to harbour a malignant disease.

Our study succeeds in demonstrating that the MTNS+ can be helpful in patients with benign USFNAs, but not as precise as when assessing indeterminate nodules. The 101 patients with benign USFNA in our study had a mean MTNS+ of 6.83 (±2.31), a value that predicts a risk of malignancy between 25 and 33 % (Table 1). Although this predicted range overestimates the true 15.8 % rate found in our study (Tables 2 and 3), both values, in support of recent studies, prove to be significantly higher than the 0–5 % stated by the ATA guidelines and the Bethesda classification [3, 9]. This leads to the question of whether an additional USFNA parameter in which points are subtracted for patients with Bethesda II USFNA results should be added to the MTNS+ in order to improve its accuracy. New studies are currently underway to answer this question.

With a statistically significant MTNS+ mean difference between malignant and benign nodules, it is clear that malignant nodules tend to have a higher MTNS+ score than benign nodules. In addition, the odds ratio of 1.34 suggests that for every increase in 1 point of MTNS+, there is an increase of 34 % in odds of malignancy for this patient population. When adjusted for nodule size in our bivariate regression analysis, the odds ratio for MTNS+ increases to 1.52 and remains statistically significant.

Williams et al., one of the only other Canadian-based studies, had also reported a falsely benign rate of 16 % [2]. Our equal malignancy rate supports their recommendation that physicians should consider the malignancy rate for the particular patient population being treated rather than solely relying on generalized literature values based on cytology [2]. At our institution it is uncommon for patients with benign USFNA’s to undergo surgery. Only patients with a high index of suspicion using the MTNS+ had surgery and were included in this analysis. More specifically, this group of patients had either worrisome ultrasound characteristics or risk factors for thyroid cancer that were a cause for concern. The MTNS+ scoring system automatically calculates these variables and enables informed and improved communication with patients. Therefore, the decision was made to operate despite the USFNA results. Other factors that may have contributed in certain cases are the presence of anxiety, compressive symptoms, or if the patient feels that the risk is too high to leave the nodule there. As a result, the true malignancy rate of patients who did not undergo surgery remains unknown. Therefore, our study does not contradict the commonly quoted 0–3 % malignancy rate of benign USFNAs stated by the Bethesda Classification [9]. In addition, the selection bias when selecting thyroidectomy patients is inevitable in surgical studies. It may also differ from one institution to another since there is still no consensus and no universal algorithm used to increase suspicion index for patients with benign USFNAs.

Large nodule size has long been proposed as a risk factor for malignancy of thyroid nodules. This holds true for patients with benign USFNAs, as previous findings have shown falsely benign rates ranging from 10.4 to 17 % [14–17] in nodules larger than 3 or 4 cm, depending on the study. Our study only supports these findings for nodules above 4 cm in size, although the malignancy rate of 7.92 % was slightly lower than that found in other studies. Only 1.98 % of nodules between 3 and 3.9 cm were malignant, which is lower than that of smaller and larger nodules (Fig. 2). This could be explained by the small number of patients in each nodule size category. However, with a weak ROC curve (Fig. 3), and since 44.58 % of nodules were above 4 cm and benign on final pathology (Fig. 2), it is clear that nodule size is a poor predictor of malignancy when used alone for patients with benign USFNAs.

The major limitation of this study is the selection bias. All patients underwent thyroidectomy based on higher clinical suspicion of malignancy using the MTNS+. This leaves the true malignancy rate unknown for patients who did not undergo surgery. Unfortunately, this is the case for research involving surgical patients.

## Conclusion

With the uncertain malignancy rate of benign USFNAs, the multitude of possible risk factors and the presence of a spectrum of management alternatives (observation alone, to re-biopsy and even thyroidectomy), the ensuing discussion with the patient is often confusing, and leads to further anxiety. MTNS+ appeases this problem as it allows the physician to describe the risk of malignancy to the patient as an individualized percentage, thus simplifying communication [1]. While MTNS+ in patients with benign USFNAs plays a role in determining the management in patients with benign thyroid nodules, it is less powerful than USFNAs with indeterminate nodules.

## Abbreviations

USFNA: ultrasound guided fine needle aspiration
MTNS+: McGill Thyroid Nodule Score +
JGH: Jewish General Hospital
MUHC: McGill University Health Centre
ETE: extrathyroidal extension
ATA: American Thyroid Association
AACE: American Association of Clinical Endocrinologists
ETA: European Thyroid Association
PET: positron emission tomography
